# Temporal and age-structured analysis of Mpox spread in the 2022 Global outbreak: data-assimilation insights for epidemic control

**DOI:** 10.1186/s40249-025-01369-7

**Published:** 2025-10-09

**Authors:** Tao Li, Xiaohao Guo, Xiaoli Wang, Tianmu Chen

**Affiliations:** 1 State Key Laboratory of Vaccines for Infectious Diseases, Xiang An Biomedicine Laboratory, State Key Laboratory of Molecular Vaccinology and Molecular Diagnostics, National Innovation Platform for Industry-Education Integration in Vaccine Research, School of Public Health, Xiamen University, No. 4221-117, Xiang’an South Road, Xiang’an District, Xiamen, Fujian Province China; 2 Beijing Center for Disease Prevention and Control, No. 16, Hepingli Middle Street, Dongcheng District, Beijing China

**Keywords:** Mpox, Transmission dynamics, Compartmental model, Age structure, Data assimilation

## Abstract

**Background:**

The global outbreak of mpox that began in 2022 caused sustained human-to-human transmission and demonstrates distinct epidemiological characteristics compared to previous outbreaks. Our aim is to quantify temporal variation of mpox transmissibility within or between age groups and evaluate the effectiveness of interventions in real time.

**Methods:**

The data used in this study is sourced from publicly available mpox confirmed cases data provided by WHO. We divided population into four age groups and constructed a transmission dynamics model with age structure of the population. And we estimated the transmissibilities of the monkeypox virus within or between age groups in real time by assimilation of global surveillance data from WHO, and performed intervention simulations in different scenarios we set up.

**Results:**

The effective reproduction number of mpox in the 18–44 age group is significantly higher than in other age groups, and it initially experiences a rapid increase, enters a phase of steady decrease after reaching a certain point [${R}_{22}=1.33$, 95% credible interval (CrI): 1.10–1.56]. Earlier implementation of interventions yields both superior effectiveness and greater cost-efficiency. Emergency vaccination for whole population initiated on June 15, 2022 reduced cumulative infections by 67.43% (95% CrI: 62.15–72.71) at only 40% coverage, whereas vaccination starting August 1, 2022 achieved only 47.86% (95% CrI: 42.70–53.01) reduction at 90% coverage. And high-risk-targeted and population-wide interventions showed limited effectiveness differences. Case management (3-day mean infectious period) initiated on July 15 achieved cumulative infection reductions of 59.84% (95% CrI: 54.28–65.40) when targeting the whole population, compared to 56.80% (95% CrI: 49.24–64.35) reduction when targeting only high-risk groups (aged 18–44 years).

**Conclusions:**

The transmissibility of mpox within the 18–44 age group follows a distinct pattern of rapid growth—slow decline not observed to the same extent in other age groups. Real-time estimation of mpox transmissibility within or between age groups helps us to understand the dynamic process of mpox interpersonal transmission better and evaluate the effect of various interventions in real world more promptly.

**Graphical Abstract:**

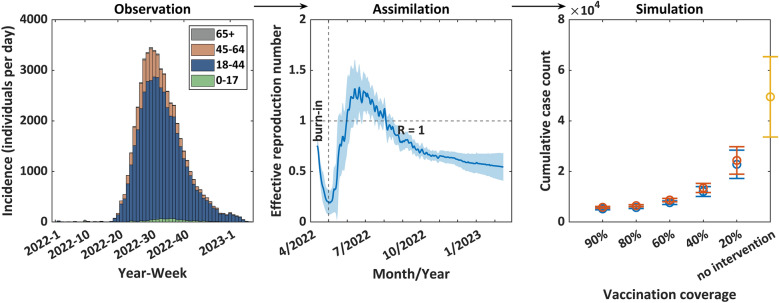

**Supplementary Information:**

The online version contains supplementary material available at 10.1186/s40249-025-01369-7.

## Background

In 2022, the World Health Organization (WHO) reported multiple outbreaks of mpox in several nations, which caused widespread transmission throughout Europe and the Americas and reached over 100 countries in all regions of the world [1]. Mpox, as a rare zoonotic disease, used to be prevalent only in Africa [2]. But in the global outbreak that began in 2022, a large proportion of the cases were reported by countries without previous records of mpox transmission, which is the first time that cases and sustained transmission chains have been reported in countries without direct or indirect epidemiological links to areas of West or Central Africa [3]. This outbreak demonstrates distinct epidemiological characteristics compared to previous outbreaks, sustained human-to-human transmission has been observed, particularly affecting men in the 18–44 age group who self-identified as men who have sex with men (MSM) [3–6]. WHO classified the monkeypox virus (MPXV) in this outbreak as clade IIb, while clade IIa denoted the non-outbreak clade [7]. On 23 July 2022, the WHO declared this global mpox outbreak a Public Health Emergency of International Concern (PHEIC), then on 11 May 2023, the WHO cancelled the PHEIC declaration.

The transmission dynamics of mpox during the global outbreak that began in 2022 exhibited systematic temporal variation, attributable to many factors such as adaptive behaviour change in affected sexual networks, naturally derived immunity and vaccination among population at high risk of mpox [8, 9]. Modelling studies from multiple affected regions (USA, Canada and Latin America) indicate that behavioural adaptations among affected communities may have substantially reduced transmission of MPXV clade IIb during the 2022 outbreak [10–12]. But few studies have systematically assessed temporal variations in mpox transmissibility based on empirical data, and current modelling studies demonstrate substantial methodological heterogeneity in their parameterization of mpox transmission dynamics, particularly regarding assumptions about behavioural adaptations in population at high risk [13–18]. And existing studies have primarily relied on aggregated case counts or assumed homogeneous mixing across age groups, potentially obscuring age-specific transmission dynamics [19, 20]. Given that adaptations to sexual behaviour due to mpox were widespread, dynamic and varied significantly across different age groups, incorporating real data to accurately estimate age-stratified temporal variations in mpox transmissibility is required.

Therefore, in this study we aim to estimate the temporal variation of mpox transmissibility within or between age groups by data assimilation, with particular focus on 18–44 age group. Through assimilation of mpox surveillance data, we seek to provide new insights into transmission dynamics of mpox in the global outbreak that began in 2022.

## Methods

### Data source

The data used for assimilation in our study is sourced from publicly available mpox confirmed cases data provided by the World Health Organization (The data set can be accessed via the following link: https://worldhealthorg.shinyapps.io/mpx_global/). Detailed information on the data is given in the Sect. "Data source" of Additional file 1. We downloaded the data on 1 September 2023, used it as the basis for our analysis, and the data source has been updated since then until now.

### Model construction

We constructed a susceptible-exposed-infectious-recovered (SEIR) transmission dynamics model with age structure based on the natural history of mpox. We divided the population into four age groups: 0–17 years old (group 1), 18–44 years old (group 2), 45–64 years old (group 3) and 65+ years old (group 4), which is consistent with the grouping of raw data from WHO. Then, we divided the population of each age group into four compartments: susceptible population, exposed population, infectious population, and recovered or removed population. Detailed descriptions of the model assumptions, ordinary differential equations (ODEs), and diagram of the model architecture can be found in the Sect. "Model construction" of Additional file 1. Table 1 summarizes all symbols and their corresponding definitions used in the model and throughout this paper.

**Table 1:** Symbols used in the model and paper, and their definitions

Symbol	Definition
${S}_{i}$	The number of susceptible individuals in age group *i*
${E}_{i}$	The number of exposed individuals in age group *i*
${I}_{i}$	The number of infectious individuals in age group *i*
${R}_{i}$	The number of removed or recovered individuals in age group *i*
${N}_{i}$	The total population in age group i
${\beta }_{ij}$	Transmission rate coefficient from infectious individuals in Age group *i* to susceptible individuals in age group *j*
$\omega$	Incidence rate coefficient (reciprocal of the mean incubation period)
$\gamma$	Recovery rate coefficient (reciprocal of the mean infectious period)
${R}_{ij}$	The expectation of secondary infections that an infected individual in the age group *i* will produce in the susceptible population of the age group *j* during the infectious period
${R}_{eff}$	The expectation of secondary infections that an infected individual in arbitrary age group will produce in the entire population during its infectious period

## Parameter estimation

Our estimation of empirical probability distributions for mpox incubation and infectious periods revealed median durations of 7.19 days [interquartile range (IQR): 6.12–8.47] and 7 days (IQR: 4–10) respectively. The specific probability distributions and estimation methodologies are detailed in the Sect. 3.1 of Additional file 1. The key parameters, transmission rate coefficients (${\beta }_{ij}$), were estimated through data assimilation, a framework that systematically combines mathematical models with observational data while accounting for uncertainty in both components. Specifically, we implemented the Ensemble Kalman Filter (EnKF), a sequential Monte Carlo method that approximates the probability distribution of model states and parameters through an ensemble of realizations [21]. Unlike least-squares, maximum likelihood, or piecewise fitting methods, the EnKF continuously updates parameter estimates as new surveillance data becomes available, capturing temporal variations in transmission dynamics that would be obscured by batch-processing approaches. And through the model's physical constraints, the EnKF can generate plausible estimates even during periods of sparse or noisy reporting. A comprehensive description of the EnKF implementation is given in the Sect. 3.2 of Additional file 1. Based on the estimated ${\beta }_{ij}$, we calculated the effective reproduction number of mpox, and its definition and derivation procedure are provided in the Sect. "Sensitivity analysis" of Additional file 1.

## Sensitivity analysis

A sensitivity analysis was conducted through Latin Hypercube Sampling (LHS) for the parameters (incubation and infectious periods, initial conditions) in the model. Our analysis reveals that several estimates exhibited sensitivity to variations in the infectious period. Detailed information about the sensitivity analysis and complete results are provided in the Sect. "Intervention simulation" of Additional file 1.

## Intervention simulation

We categorized the interventions into three types: emergency vaccination, interrupting transmission routes (such as contact reduction and safe sexual practices), and case management (including contact tracing and prompt isolation/treatment). Then we simulated the implementation of these three intervention categories under varying initiation timings, implementation intensities, and target populations, evaluating their effectiveness through cumulative case count, relative reduction in cumulative case count, time of incidence peak and advancement or delay of incidence peak (See Additional file 2 for complete details).

## Software and code

Model construction, parameter estimation, sensitivity analysis and intervention simulation were all performed on MATLAB R2024b. All model codes and analysis scripts, including the parameter file, data preprocessing routines and simulation procedures, have been deposited in the GitHub repository (https://github.com/WDZHITIETOU/Mpox_EnKF.git) with open-access under MIT License to ensure reproducibility.

## Results

### Epidemiological characteristics of 2022 mpox outbreak

**Fig. 1:**
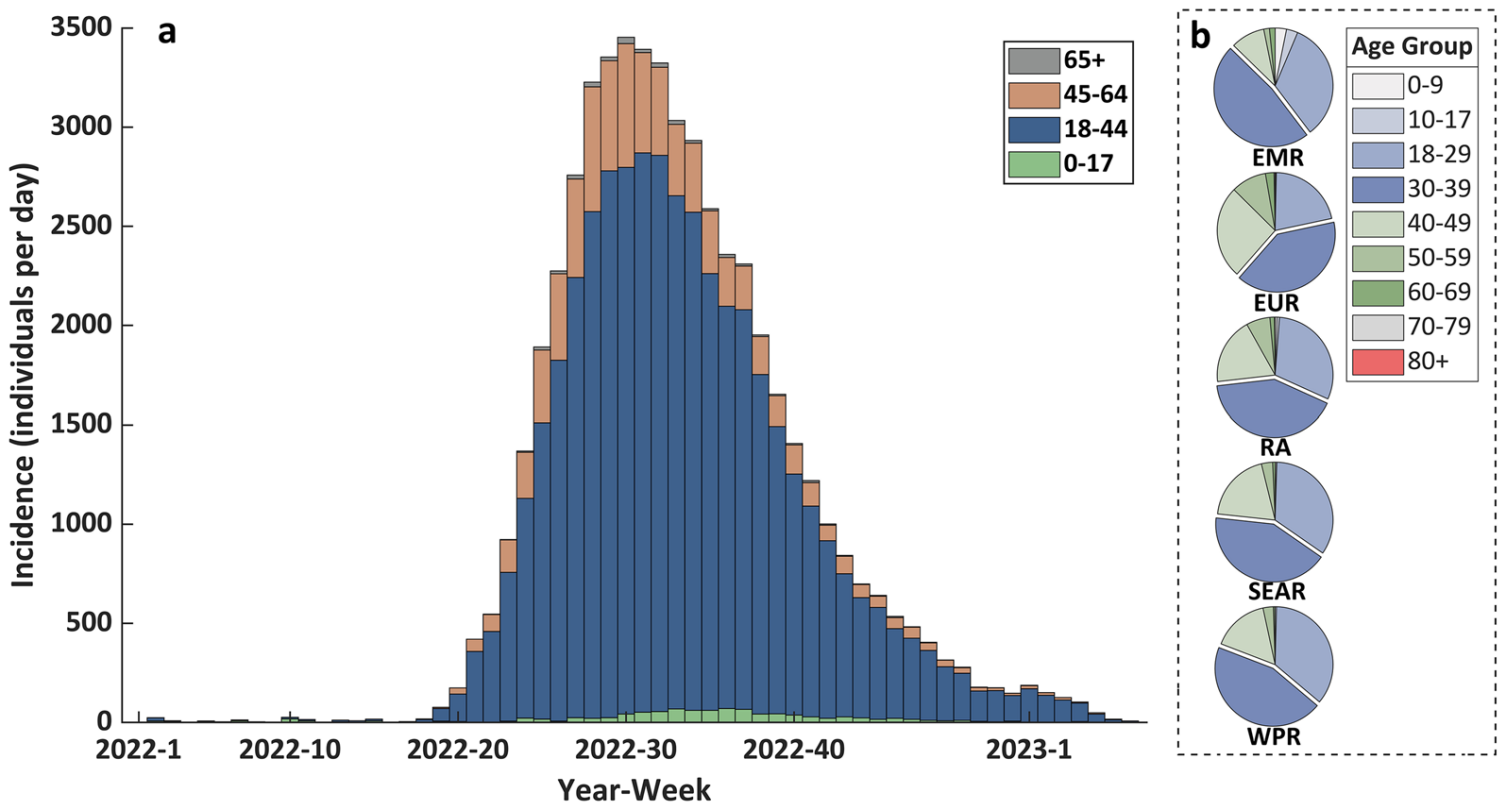
Temporal, and age distribution of mpox cases in the global outbreak that began in 2022. **a**. illustrates the global mpox epidemic curve from January 1, 2022, to February 15, 2023, where the vertical axis reflects the number of new cases reported daily and the horizontal axis denotes the year and week, such as 2022–1 representing the first week of 2022. Week 1 of the year starts on January 1st, with every following week of the year starting on a Sunday. Different colours in the bar chart denote different age groups. **b**. presents five pie charts that describe the age distribution of confirmed mpox cases across five regions of the world excluding Africa. EMR represents the Eastern Mediterranean Region, EUR represents the European Region, RA represents the Region of the Americas, SEAR represents the South-East Asia Region, and WPR represents the Western Pacific Region. A slice offset from the rest of the pie chart represents the age group with the largest proportion

The epidemiological profile of the mpox outbreak from January 1, 2022, to February 15, 2023 was illustrated on Fig. 1. The United States has reported the highest number of confirmed mpox cases, followed by Brazil, and European nations including Spain and France. During this period, a total of 53,003 confirmed cases of mpox were reported worldwide, of which there were 944 cases (1.78%) in the age group 0–17, 44,404 cases (83. 78%) in the 18–44 age group, 7347 cases (13.86%) in the 45–64 age group, and 308 cases (0.58%) in the age group 64 + . In early May 2022, there was a noticeable increase in the number of new cases, reaching a peak in mid-July and gradually decreasing to near-zero by mid-February 2023. The age distribution of mpox cases was remarkably similar in the five regions outside of Africa, with the majority of cases found in the 30–39 age group. This was followed by the 18–29 and 40–49 age groups, which together accounted for more than 80% of the reported cases. While in Africa the highest number of cases with mpox are aged 0–9 years, followed by 18–29 years and 30–39 years [3].

### Varying age-specific transmissibility of MPXV

The observation data from 12 April 2022 to 15 February 2023 are used for assimilation. To address the bias between the initial ensemble distribution in EnKF and the true system state, we discard the state estimates from the first 19 iterations, similar to the burn-in concept in Markov Chain Monte Carlo (MCMC) [22], so the first 19 days are considered as burn-in period. The EnKF demonstrates reliable performance, and detailed information is provided in the Sect. 3.2 of Additional file 1.

**Fig. 2:**
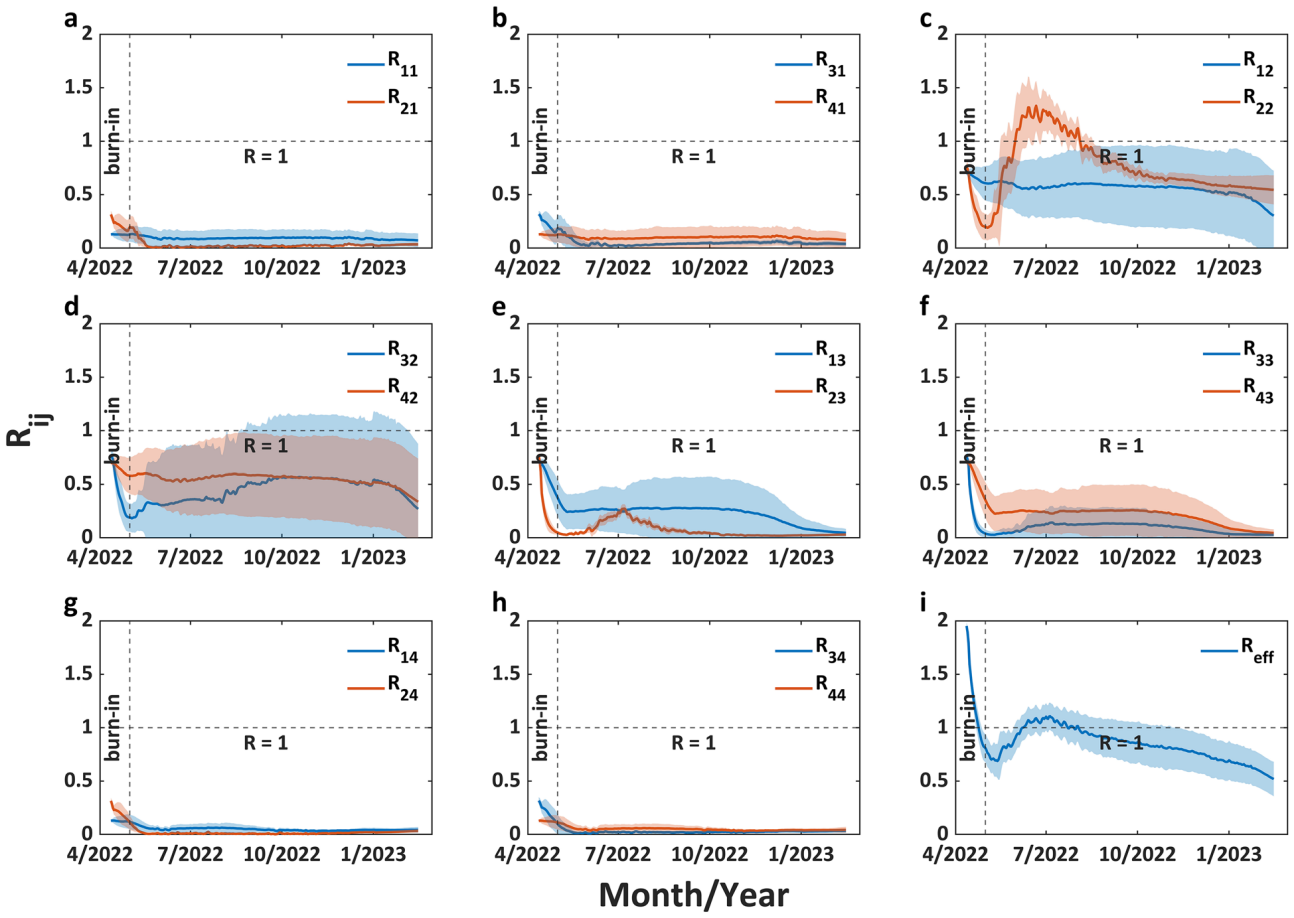
Distributions of instantaneous effective reproduction numbers within or between age groups. **a**. to **h**. illustrate the distributions of ${R}_{ij}$ (the expectation of secondary infections generated by an infected individual from the age group $i$ in the susceptible population of the age group $j$ throughout the infectious period) across all discrete time intervals. **i**. illustrates the distribution of ${R}_{eff}$ (the expectation of secondary infections that one infected individual of an arbitrary age group will produce in the entire population throughout its infectious period) across all discrete time intervals. The segment preceding the vertical dashed line indicates the burn-in period, the horizontal dashed line marks the threshold where the effective reproduction number is equal to 1. The solid line represents the mean, and the light shaded region represents the 95% credible interval

**Fig. 3:**
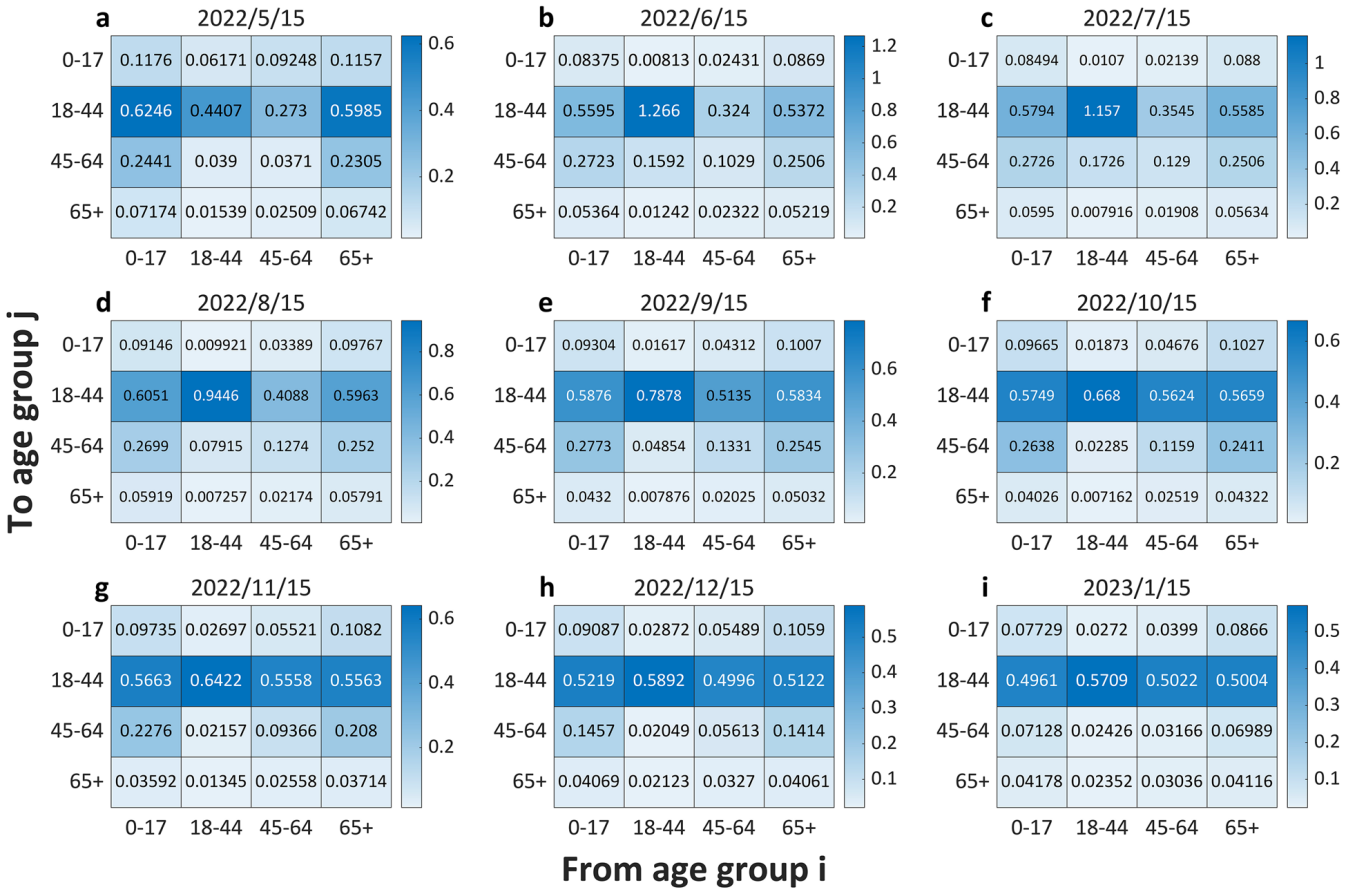
Heat maps of the interactive ${R}_{ij}$ between age groups at different dates. **a**. to **i**. illustrate matrix of ${R}_{ij}$ represented by a heat map on 15 May 2022, 15 June 2022, 15 July 2022, 15 August 2022, 15 September 2022, 15 October 2022, 15 November 2022, 15 December 2022 and 15 January 2023 respectively. In each heat map, the element in row j and column i represents the mean of ${R}_{ij}$ (the expectation of secondary infections generated by an infected individual from the age group $i$ in the susceptible population of the age group $j$ throughout the infectious period), and gradual color gradient reflects value scale

As shown in Fig. 2, ${R}_{22}$ exhibits a rapid initial increase, reaching its peak [${R}_{22}=1.33$, 95% credible interval (CrI): 1.10–1.56] on 21 June 2022, followed by a gradual decline to 0.65 (95% CrI: 0.60–0.70) by 1 November 2022. Subsequently, ${R}_{22}$ stabilizes around 0.59 (95% CrI: 0.49–0.68) after 1 January 2023. ${R}_{23}$ has a similar epidemiological pattern to ${R}_{22}$, except that ${R}_{23}$ exhibits relatively small variations. ${R}_{23}$ peaked on 7 July 2022 (${R}_{23}=0.27$, 95% CrI: 0.23–0.32), declined to 0.025 (95% CrI: 0.015–0.035) by 28 October 2022, and subsequently stabilized at approximately 0.023 (95% CrI: 0.016–0.031). ${R}_{32}$ and ${R}_{42}$ consistently maintained values around 0.5 throughout the study period, but both showed relatively wide 95% credible intervals, with lower limits approaching 0 and upper limits around 1. The other interactive ${R}_{ij}$ maintained values between 0 and 0.5 with no observable patterns of change. The effective reproduction number in entire population (${R}_{eff}$) exhibits an initial increase, reaching its peak (${R}_{eff}=1.11$, 95% CrI: 0.98–1.23) on 5 July 2022, followed by a sustained decline to 0.52 (95% CrI: 0.36–0.68) by 15 February 2023. Figure 3 more intuitively illustrates the mutual influences among different age groups. Although mpox transmissibility within the 18–44 age group is significantly higher than other age groups (effective reproduction number within this age group reached 1.27 by 15 June 2022, whereas all other groups remained below 0.56), infectious individuals in this group demonstrate limited cross-group transmission potential, with little infection spread to susceptible individuals in other age groups. Interestingly, infectious individuals from other age groups were more likely to transmit the infection to susceptible individuals aged 18–44.

### Comparative results of different intervention simulations

As of September 2022, the effective reproduction numbers of mpox (both within and between age groups) had dropped below 0.8, indicating that the combined effects of global intervention measures and adaptive behavioural changes among susceptible populations had successfully mitigated further disease transmission. Consequently, the three-month period from June through August 2022 was selected as the pivotal window for implementing mpox interventions.

**Fig. 4:**
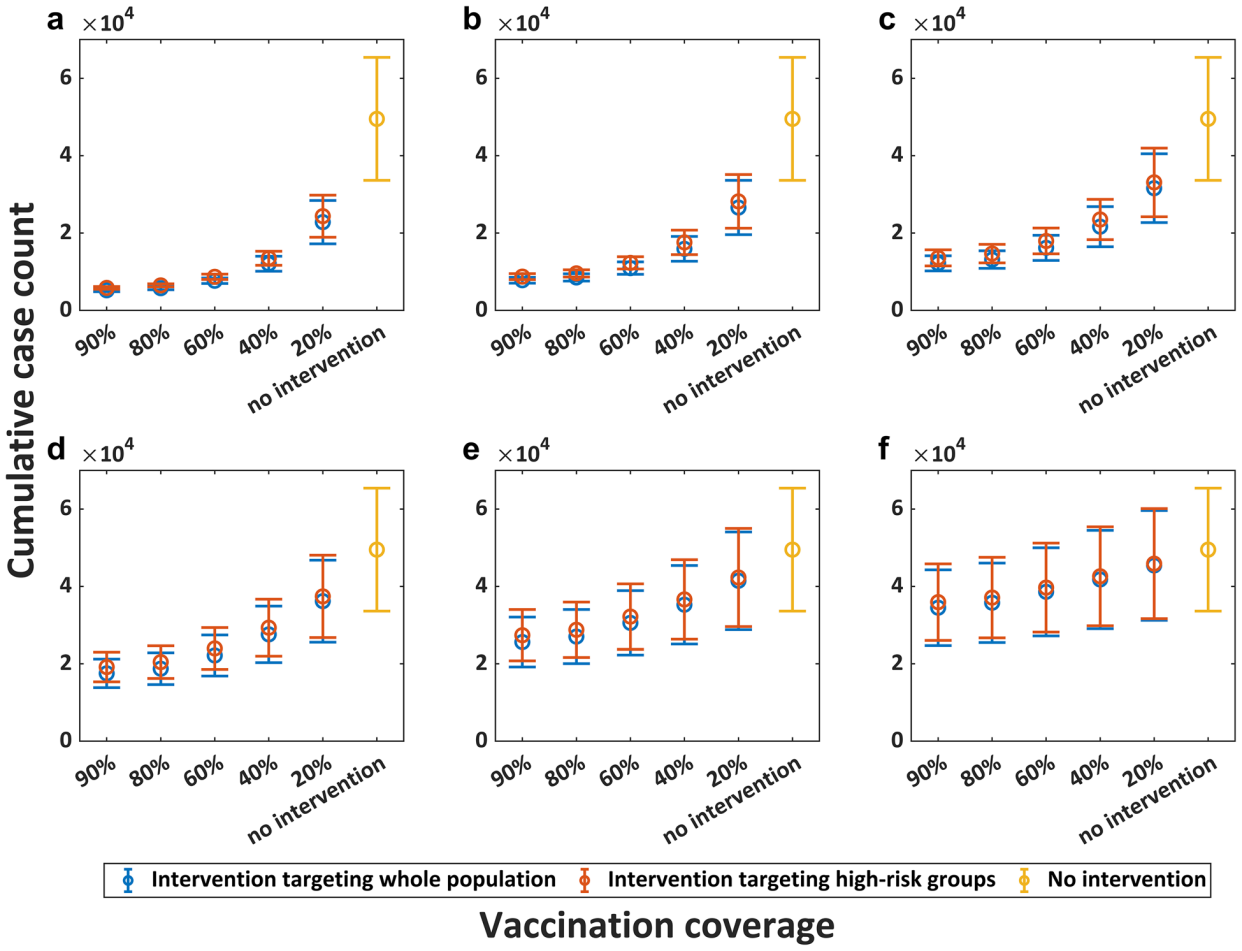
Intervention effect of emergency vaccination. **a**. to **f**. represent different intervention initiation dates: June 1, June 15, July 1, July 15, August 1, and August 15, 2022, respectively. The horizontal axis represents the vaccination coverage, while the vertical axis represents the corresponding cumulative case count. Circles represent mean cumulative case counts, with upper and lower horizontal lines indicating the 95% credible interval (CrI) upper and lower limits, respectively. Blue represents emergency vaccination for the whole population, while red indicates vaccination targeting only high-risk groups (aged 18–44 years)

**Fig. 5:**
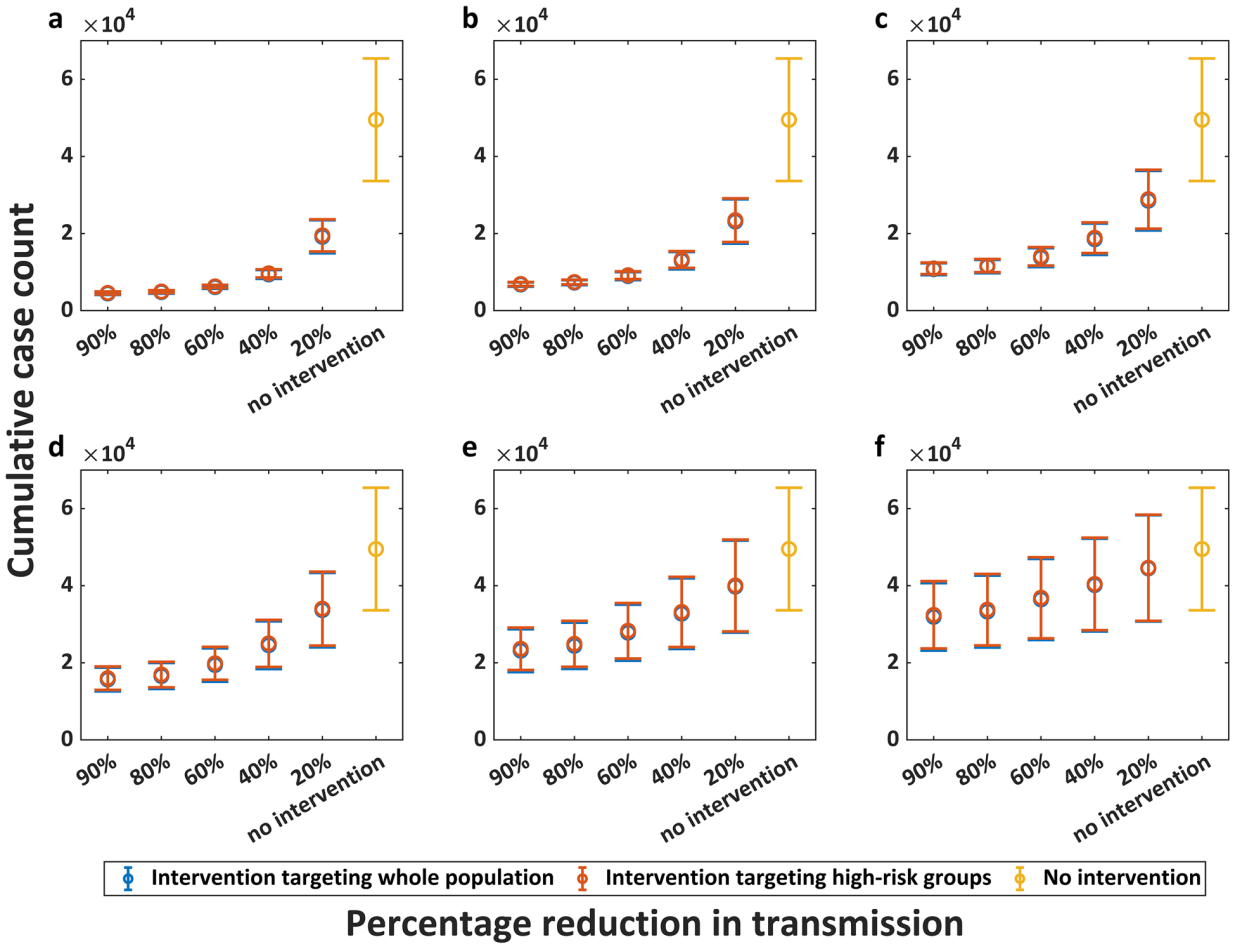
Intervention effect of interrupting transmission routes. **a**. to **f**. represent different intervention initiation dates: June 1, June 15, July 1, July 15, August 1, and August 15, 2022, respectively. The horizontal axis represents the percentage reduction in transmission, while the vertical axis represents the corresponding cumulative case count. Circles represent mean cumulative case counts, with upper and lower horizontal lines indicating the 95% credible interval (CrI) upper and lower limits, respectively. Blue represents interrupting transmission routes among the whole population, while red indicates interrupting transmission routes only among high-risk groups (aged 18–44 years)

**Fig. 6:**
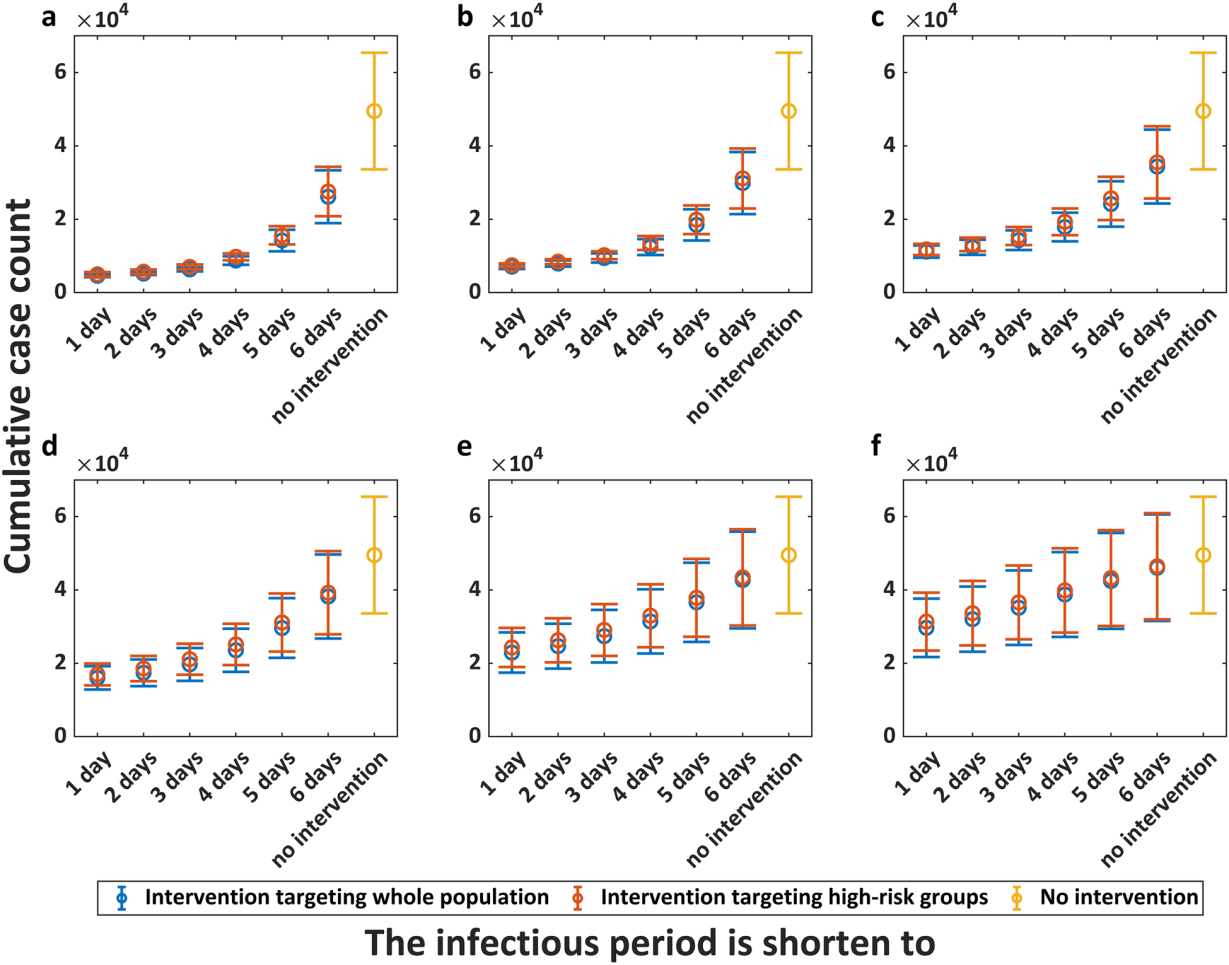
Intervention effect of case management. **a**. to **f**. represent different intervention initiation dates: June 1, June 15, July 1, July 15, August 1, and August 15, 2022, respectively. The horizontal axis represents the shortened infectious period, while the vertical axis represents the corresponding cumulative case count. Circles represent mean cumulative case counts, with upper and lower horizontal lines indicating the 95% credible interval (CrI) upper and lower limits, respectively. Blue indicates management of all cases, while red represents management focusing only on key cases (aged 18–44 years)

Figures 4, 5 and 6 present the intervention effectiveness of emergency vaccination, interrupting transmission routes, and case management, respectively. First, the results demonstrates that earlier implementation of interventions yields both superior effectiveness and greater cost-efficiency. Emergency vaccination for whole population initiated on June 15, 2022 reduced cumulative infections by 83.83% (95% CrI: 78.53–89.13) at 90% coverage and by 67.43% (95% CrI: 62.15–72.71) at 40% coverage, whereas vaccination starting August 1, 2022 achieved 47.86% (95% CrI: 42.70–53.01) reduction at 90% coverage and only 28.5% (95% CrI: 25.12–31.88) reduction at 40% coverage. Second, our analysis reveals diminishing marginal returns of intervention intensity—beyond certain thresholds, additional intensification yields limited benefit. Starting from July 1, 2022, interrupting 90% of transmission among whole population could reduce cumulative infections by 77.83% (95CrI: 72.20–83.47), interrupting 80% by 76.45% (95% CrI: 70.78–82.11), interrupting 60% by 71.79% (95% CrI: 66.16–77.42), interrupting 40% by 62.19% (95% CrI: 56.87–67.51), and interrupting 20% by 42.07% (95% CrI: 38.12–46.02). Third, high-risk-targeted and population-wide interventions showed limited effectiveness differences. Case management initiated on July 15 achieved cumulative infection reductions of 59.84% (95% CrI: 54.28–65.40) (3-day mean infectious period) and 52.08% (95% CrI: 47.09–57.07) (4-day mean infectious period) when targeting the whole population, compared to 56.80% (95% CrI: 49.24–64.35) and 48.68% (95% CrI: 41.55–55.81) reduction when targeting only high-risk groups (aged 18–44 years).

## Discussion

Through the transmission dynamic model with age structure, our study simulated the interpersonal transmission of MPXV in the global outbreak began in 2022. Significantly different from previous studies, we estimated the transmission rate coefficient of mpox within or between age groups in real-time by data assimilation for the first time. This allows us to observe the epidemics process of mpox dynamically and estimate changes in mpox transmissibility in real time without relying on some assumptions about the mechanisms of change. The transmissibility of mpox is influenced by many factors, such as community interventions, vaccinations, behavioural changes, etc*.* We quantified the combined effect of these factors on mpox transmissibility by assimilating surveillance data, so that we could evaluate the effectiveness of interventions in real time and optimize their implementation promptly.

In terms of reproduction number, the findings of our study are generally in agreement with previous studies [23, 24]. There was a significant increase compared with mpox outbreaks in Africa prior to the additional upsurge of cases in 2023 and 2024 [25, 26]. However, limitations in completeness and timeliness of case surveillance data will lead to an underestimation of the transmissibility of mpox, the actual coefficient of transmission rate (${\beta }_{ij}$) and effective reproduction number (${R}_{ij}$) may be higher than our results.

Our results indicate that in age group 18–44 years, the transmissibility of mpox initially experiences a rapid increase, enters a phase of steady decrease after reaching a certain point, which is possibly related to the contact patterns and behavioural characteristics of this population. In the 2022 outbreak of mpox, sexual contact has become the main route of transmission. According to data from WHO [3], among 18,980 cases with confirmed transmission information, 15,588 cases were transmitted by sexual contact. And male-male sexual behaviour was identified as the predominant mode of sexual contact. Among the cases with known information on sexual orientation, 84.1% (25,763/30,642) were confirmed to be men who have sex with men. Baral et al*.* estimated that approximately 60% of men who have sex with men are between 18 and 34 years old based on statistical data from Facebook users [27]. The population in this age group typically has a more active social life and more frequent interpersonal contacts, making them more prone to contact with the virus and spreading it to others. And many men who have sex with men affected in the global mpox outbreak reported contact with several (or multiple) sexual partners. Early epidemiological investigations found that MPXV clade IIb transmission in non-endemic countries occurred predominantly through densely interconnected sexual networks among men who have sex with men [28], which may contribute to the rapid rise in mpox transmissibility during the early epidemic phase. After rapid epidemic growth between May and August, 2022, a decline in confirmed mpox case incidence in Europe and the Americas was observed towards the end of 2022 with low-level transmission in these regions continuing [3], which may be due to behavioural change and naturally derived immunity [8]. This trend aligns with our estimated temporal dynamics of mpox transmissibility in the 18–44 age groups.

While our age-structured model provides important insights into mpox transmission patterns, several limitations regarding the use of age-based proxies in the context of mpox’s sexual transmission dynamics should be acknowledged. First, epidemiological studies have demonstrated that mpox transmission during the 2022 outbreak was predominantly concentrated within dense sexual networks of MSM, particularly those with multiple partners or participation in group sex events [4], characteristics that are not uniformly distributed across age groups nor perfectly correlated with age. Second, our approach may inadvertently conflate age-related biological susceptibility with behavioural risk factors. Younger individuals may have higher partner acquisition rates [29], while older MSM might have more stable partnerships but potentially greater connectivity within sexual networks [29, 30]. The lack of explicit behavioural stratification means our model cannot capture these nuanced transmission dynamics. Third, the assumption of homogeneous mixing within age groups fails to represent the core-periphery structure observed in real-world MSM sexual networks [10], where a small subset of highly connected individuals drives disproportionate transmission. Fourth, our model does not take into account the impact of vaccination [31] and differences in the effects of different potential modes of transmission on the spread of mpox (sexual, other forms of direct contact including respiratory secretions, contact with fomites, exposure in the health care setting, etc.). These limitations suggest that while age structure provides a useful first approximation, future modelling efforts should incorporate both demographic and behavioural stratifications (consider stratifying population by age, gender [32], sexual orientation and vaccination, categorizing contact patterns into high-risk contacts and general contacts) to better inform targeted prevention strategies. Our findings should therefore be interpreted as representing general population trends rather than precise estimates of transmission within high-risk subgroups.

Based on the intervention simulation results, we recommend prioritizing control measures for the 18–44 age group, particularly during the two-month period of June and July 2022. But our modelling approach incorporates several simplifying assumptions regarding intervention simulation. First, we assumed interventions (e.g., emergency vaccination, case management) would be adopted immediately and homogeneously across the population—a scenario rarely achieved in practice due to logistical delays (e.g., resource allocation, public compliance) and spatial heterogeneity in adherence (e.g., urban vs. rural areas). Second, our model did not incorporate dynamic adjustments in intervention implementation processes. Third, our model does not account for behavioural feedback (e.g., fatigue leading to reduced compliance over time) or socioeconomic disparities in access to interventions. While this assumption facilitates direct comparison of intervention effect, real-world deployment would likely exhibit phased rollout and variable uptake, as observed in Australia [33]. These limitations highlight a trade-off between theoretical generalizability and practical feasibility. Future work could integrate dynamic adherence patterns or agent-based frameworks to better capture these nuances.

## Conclusions

In order to quantify differences in the risk of infection among different age groups during the 2022 mpox outbreak, our study constructed a multi-group SEIR transmission dynamics model and estimated the temporal variation of mpox transmissibility by data assimilation for the first time. Our results indicate that the transmissibility of mpox is significantly higher in the 18–44 age group than in other age groups, and follows a distinct pattern of rapid growth—slow decline not observed to the same extent in other age groups. Reducing mpox transmission among people aged 18–44 years will play a crucial role in controlling the spread, and an earlier response will lead to more effective prevention and control effects. Real-time estimation of mpox transmissibility within or between age groups helps us to understand the dynamic process of mpox interpersonal transmission better and evaluate the effect of various interventions in the real world more promptly. Public health agencies should optimize of the surveillance data management pipeline to better capture temporal variation of disease transmissibility, similar to our approach. Our study provides valuable information for optimizing mpox prevention and control measures and helps practitioners understand and respond to emerging infectious diseases like mpox better.

## Supplementary Information

Additional file 1. Detailed information about the methods section, and a summary table of the intervention scenarios.

Additional file 2. Comprehensive information about the results of intervention simulation.

## Data Availability

The datasets used and analysed during the current study are available from https://worldhealthorg.shinyapps.io/mpx_global/.

## Abbreviations

WHO: World Health Organization
MSM: Men who have sex with men
CrI: Credible interval
IQR: Interquartile range
EnKF: Ensemble Kalman Filter
MPXV: Monkeypox virus
MCMC: Markov Chain Monte Carlo
PHEIC: Public Health Emergency of International Concern
SEIR: Susceptible-exposed-infectious-recovered
