# Supplementary material for: Temporal and age-structured analysis of Mpox spread in the 2022 Global outbreak: data-assimilation insights for epidemic control
Source: Infect Dis Poverty. 2025 Oct 9;14:100. doi: 10.1186/s40249-025-01369-7 (PMC12512411; doi:10.1186/s40249-025-01369-7)
Supplement: Supplementary file 1 — Additional file 1. Detailed information about the methods section, and a summary table of the intervention scenarios. [file 40249_2025_1369_MOESM1_ESM.pdf]

Supplementary Document

Temporal and Age-Structured Analysis of Mpox Spread in the 2022 Global Outbreak: Data-assimilation Insights for Epidemic Control

Tao Li, Xiaohao Guo, Xiaoli Wang, Tianmu Chen

Contents

1 Source and description of the observation data 2

2 Mpox transmission model with age structure 3

3 Parameter estimation 5

3.1 Probability distribution of natural history parameters 5

3.2 Data assimilation of reported daily incidence 6

4 Effective reproduction number for assessing transmissibility 11

5 Sensitivity analysis 13

References 15

1 Source and description of the observation data

The observation data used for data assimilation in this study are sourced from publicly available mpox laboratory-confirmed cases data provided by the World Health Organization (https://worldhealthorg.shinyapps.io/mpx_global/). This data contains the following information:

1. Date of symptom onset (from 12 April 2022 to 15 February 2023), date of lab or clinical diagnosis (if date of symptom onset is not available), date of reporting (if date of symptom onset and date of diagnosis are not available);

2. Age groups (including 0–17 year olds, 18–44 year olds, 45–64 year olds and 65+ year olds);

3. Number of daily new cases.

It is evident that the raw data exhibits significant measurement noise, so we smoothed the raw data using the moving average method and the size of window is 5 (Figure S1).

The World Health Organization relies on direct reporting from member countries to collect mpox cases data and has taken measures to ensure its accuracy and reliability. However, all counts are subject to variations in case detection, definitions, laboratory testing, and reporting strategies between countries, states, and territories. In the report of WHO [1], it is mentioned that during the global mpox outbreaks in 2022–23, the frequency of case reporting was not high, and there may have been delays between case detection and reporting, especially in African regions, where there are significant gaps between reported confirmed cases and suspected cases.

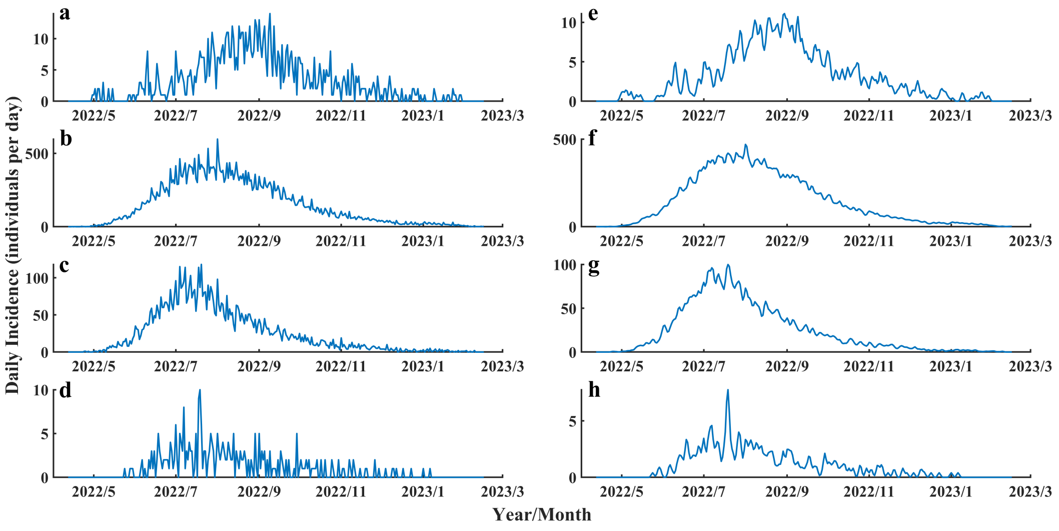

Figure S1: Raw and smoothed data. The horizontal axis indicates the date and the vertical axis indicates the number of new cases reported per day. Subfigures a, b, c, and d represent raw data for age groups 0–17, 18–44, 45–64, and 65+ respectively. Subfigures e, f, g, and h represent smoothed data for age groups 0–17, 18–44, 45–64, and 65+ respectively.

2 Mpox transmission model with age structure

To quantitatively assess the transmissibility of mpox within or between age groups, we constructed a transmission dynamics model with age structure based on the natural history of mpox. We divided the population into four age groups: 0–17 years old (group 1), 18–44 years old (group 2), 45–64 years old (group 3) and 65+ years old (group 4), which is consistent with the grouping of raw data from WHO. The natural history of disease refers to the pattern of development of a disease or health condition in the absence of treatment, intervention, or preventive measures [2]. In the case of mpox, infection may occur after a susceptible individual has direct or indirect contact with a person with mpox or with the virus. Infected individuals will not show symptoms immediately and need to go through an incubation period during which they are called exposed individuals. Once symptoms appear, they become infectious and remain so for a duration known as the infectious period. Once cases recover or are removed from the population due to measures such as isolation, vaccination or hospitalization, they lose their infectivity. Therefore, we divided the population of each age group into four compartments: susceptible population, exposed population, infectious population, and recovered or removed population. And we introduced the following variables: the number of susceptible individuals indicated by S, the number of exposed individuals indicated by E, the number of infectious individuals indicated by I, the number of removed or recovered individuals indicated by R; size of the total population indicated by N and the different age groups are indicated by subscripts i and j, $i, j \in \{1, 2, 3, 4\}$.

Assumptions regarding the model framework and parameter specifications are summarized as follows:

1. β_{ij} represents the transmission rate coefficient. The transmission rate, the rate of moving from compartment of susceptible population to compartment of exposed population, is directly proportional to the number of susceptible and infectious individuals and β_{ij} is its coefficient. In fact β_{ij} contains the assumption that on average an individual in age group i makes a constant number β_{ij} of contacts sufficient to transmit infection with individuals in age group j in unit time.

2. ω represents the incidence rate coefficient. Incidence rate, the rate of moving from compartment of exposed population to compartment of infectious population, is directly proportional to the number of exposed individuals. According to the properties of ordinary differential equations, the incubation period follows an exponential distribution with a mean of $\frac{1}{\omega}$, so ω is equal to reciprocal of the mean incubation period.

3. γ represents the recovery rate coefficient. Recovery rate, the rate of moving from compartment of infectious population to compartment of recovered or removed population, is directly proportional to the number of infectious individuals. Similarly to the incubation period, the infectious period follows an exponential distribution with a mean of $\frac{1}{\gamma}$, so γ is equal to the reciprocal of the mean infectious period. The infectious period here refers to the duration between the onset of symptoms and diagnosis, because it is assumed that individuals with symptoms will be required to follow isolation measures or voluntarily reduce their contact with others after being diagnosed.

4. Short-term outbreaks of disease do not take into account demographic indi-

cators such as natural mortality, birth rates, etc. Vertical mother-to-child transmission and the presence of asymptomatic infections have been ignored due to lack of evidence [3]–[5]. Compared with the fatality rate for Clade I (1%–11%) and Clade II (3%–6%) that were previously endemic in Africa [6], the fatality rate in this mpox outbreak is very low, less than 0.1%, and some of the deaths have been related to meningitis and immunocompromise [7], [8]. In addition, due to the lack of detailed data on deaths among patients with mpox, the fatality rate of the cases in different age groups was not considered in the model.

The ordinary differential equations (ODE) of age structured SEIR model are given by:

$$\begin{aligned}
\frac{dS_i}{dt} &= -\sum_{j=1}^{n} \frac{\beta_{ji} S_i I_j}{N_i} \\
\frac{dE_i}{dt} &= \sum_{j=1}^{n} \frac{\beta_{ji} S_i I_j}{N_i} - \omega E_i \\
\frac{dI_i}{dt} &= \omega E_i - \gamma I_i \\
\frac{dR_i}{dt} &= \gamma I_i,
\end{aligned} \tag{1}$$

where $n = 4$; $N_j = S_j + E_j + I_j + R_j$. Figure S2 can more clearly illustrate the flow of people between different compartments and the transmission among different age groups.

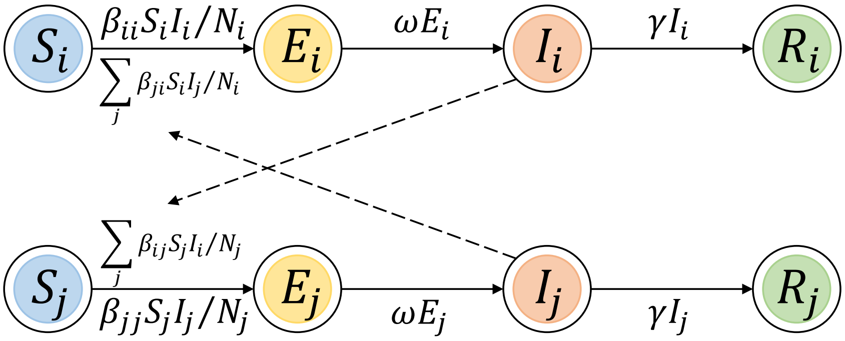

Figure S2: Age structured SEIR Model. The symbols shown in the figure have been explained in the model assumptions. Concentric circles denote compartments, solid arrows denote the direction of movement between compartments, formula positioned above and below the solid arrows denote move rates, and dashed arrows represent susceptible individuals in an age group are infected by infectious individuals in other age groups.

3 Parameter estimation

As can be seen from the assumption of the model, the parameters to be estimated include β_{ij}, ω, and γ. Estimation of ω and γ was carried out through the relationship between them and natural history parameters of mpox that these parameters are the reciprocals of the mean incubation period and the mean infectious period respectively. For the estimation of β_{ij}, we used methods of data assimilation (Table S1).

Table S1: Table of model parameters

parameter	description	unit	value*	estimation
β_{ij}	transmission rate coefficient	individuals per day	-	assimilate by observation data
$\frac{1}{\omega}$	incubation period	day	7.19 (IQR: 6.12-8.47)	kernel density
$\frac{1}{\gamma}$	infectious period	day	7 (IQR: 4-10)	piecewise linear distribution

* Here the parameter is presented using its median, lower quartiles and upper quartiles.

3.1 Probability distribution of natural history parameters

Based on the relevant literature and public data from the WHO, we estimated the empirical probability distributions of the incubation period and the infectious period. For the incubation period, we computed the kernel density estimator, which represents the estimated probability density function (pdf) of a stochastic variable. For any real values of x, the kernel density estimator's formula is given by:

$$\hat{f}_h(x) = \frac{1}{nh}\sum_{i=1}^{n} K(\frac{x - x_i}{h}), \tag{2}$$

where x_1,x_2,...,x_n are random samples from an unknown distribution, n is the sample size, $K(\cdot)$ is the kernel smoothing function, and h is the bandwidth. In order to estimate the incubation period of mpox, we collected some study findings, considered them as random samples from an unknown distribution (x_1,x_2,...,x_n), and fitted the smooth distribution based on the Gaussian kernel function by using the sample size as weights. Table S2 shows these study findings and the corresponding references.

A piecewise linear distribution estimates an overall cumulative density function (cdf) for the samples by computing the cumulative probability density value at each individual point and then linearly connecting these values to form a continuous curve. Due to the linear connection and the fact that the derivative of the cumulative probability density function is discontinuous at the connection points, the probability density function (the derivative of the cumulative probability density function) remains constant between the connection points and jumps at the connection points. In our study, we used the lower quartile (4 days), median (7 days), upper quartile (10 days) [1], minimum value (0 days) and maximum value (28 days) [24]–[26] of infectious period as individual points to calculate its piecewise linear distribution.

Table S2: Research findings and references on incubation period

Reference	Sample size	Point estimate	
		mean*	median*
Miura et al. [9]	18	8.5	
Charniga et al. [10]	40	7.6	6.4
Thornhill et al. [11]	23		7.0
Català et al. [12]	77		6.0
Tarín-Vicente et al. [13]	144		7.0
Guzzetta et al. [14]	30	9.1	
Mailhe et al. [15]	112		6.0
Moschese et al. [16]	16		11.0
Gomez-Garberi et al. [17]	14		13.0
O'Laughlin et al. [18]	527	7.0	
Angelo et al. [19]	78		8.0
Madewell et al. [20]	35	5.6	
Ward et al. [21]	54	7.8	
Besombes et al. [22]	29		7.0
Kröger et al. [23]	209	8.2	

* The unit is days.

The probability density curves of incubation period and infectious period are presented in Figure S3. The values of infectious period and incubation period in the model are medians derived from their empirical probability distributions (Table S1). Although there is a certain deviation between the empirical distributions obtained by the limited samples and the actual probability distributions of these parameters, given the lack of detailed individual case data, this deviation is acceptable.

3.2 Data assimilation of reported daily incidence

To accurately evaluate disease transmission in the model, specifically the transmission rate coefficient (β_{ij}), we have opted to use data assimilation to iteratively refine

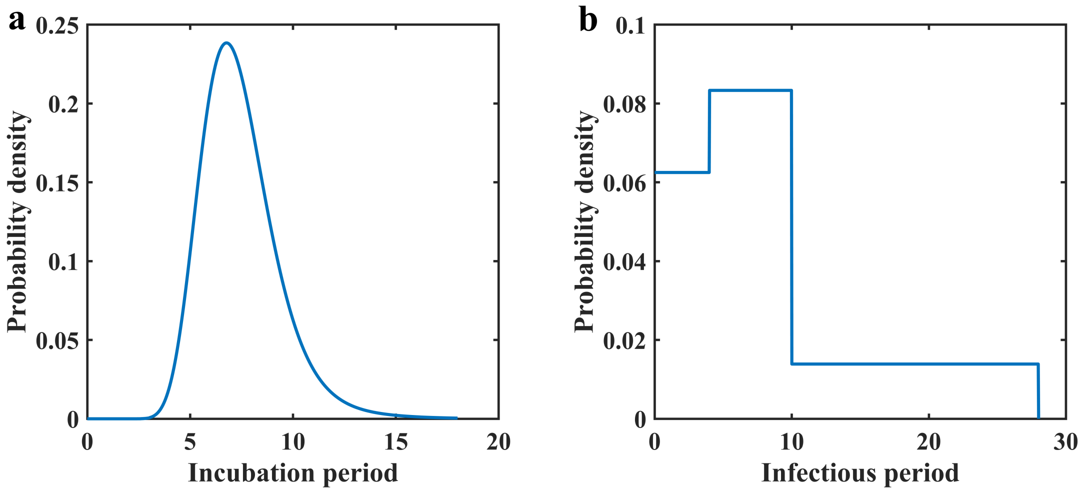

Figure S3: Probability density curves. Subfigure a indicates the probability density curve of incubation period, while subfigure b indicates the probability density curve of infectious period. The horizontal axis indicates the value of incubation or infectious period (unit: days) and the vertical axis indicates the corresponding probability density.

the estimated value of this parameter in real time. Data assimilation is a process of combining observation data with a mathematical model to improve the accuracy of dynamic system output [27]. In the section of Model construction, we used ordinary differential equations to describe the nonlinear system of disease transmission (Equation 1). Prior to data assimilation, we need to employ the State-Space Model (SSMs), a statistical framework designed to characterize the system's behaviour over time [28], to analysis this system. In SSMs, the system is represented by two sets of equations: the state equation and the observation equation. The state equation describes how the system evolves over time, while the observation equation describes how the system is observed or measured.

First, we have made the following definitions to construct the state vector: $\boldsymbol{S} \overset{def}{=} [S_1; S_2; S_3; S_4]$, $\boldsymbol{E} \overset{def}{=} [E_1; E_2; E_3; E_4]$, $\boldsymbol{I} \overset{def}{=} [I_1; I_2; I_3; I_4]$, $\boldsymbol{R} \overset{def}{=} [R_1; R_2; R_3; R_4]$, $vec(\beta_{ij}) \overset{def}{=} [\beta_{11}; \beta_{12}; \cdots; \beta_{43}; \beta_{44}]$, where ";" denotes stacking by column, and "[]" denotes forming a vector with inner elements. All of these formed the state vector:

$$\boldsymbol{x} \overset{def}{=} [\boldsymbol{S}; \boldsymbol{E}; \boldsymbol{I}; \boldsymbol{R}; vec(\beta_{ij})], \quad \boldsymbol{x} \in \mathbb{R}^{32}. \tag{3}$$

Then we used the Euler's Method to obtain the time discretization of Equation 1 and its expression using the state vector is as follows.

$$\boldsymbol{x}_t = \boldsymbol{x}_{t-1} + h_{t-1} \cdot \frac{d\boldsymbol{x}_{t-1}}{dt}, \tag{4}$$

where t denotes the t-th time step, $h_{t-1} = 1\,day$ represents step size of time. And we assumed $\frac{d}{dt}vec(\beta_{ij}) = \mathbf{0}$. This model assumption does not really reflect reality, as it has been shown that there has been a systematic change in the high-risk behaviours of susceptible populations during mpox global outbreak that began in 2022 [29], and relevant model studies have assumed the effect of behavioural change on transmission [30]. The reason for our assumption is that we would like to correct the model's prediction of the transmission rate coefficients in real time by assimilate the global surveillance data. Changing estimates of the transmission rate coefficients obtained

by data assimilation reflect the possible effects of behaviour adaptation and other factors on disease transmission.

Subsequently, by introducing noise following a normal distribution into the process described by Equation 4, we obtained the state equation:

$$\boldsymbol{x}_t = f(\boldsymbol{x}_{t-1}) + \boldsymbol{w}_t, \quad \boldsymbol{w}_t \sim \mathcal{N}_n(\boldsymbol{0}, \boldsymbol{Q}_t), \tag{5}$$

where $n = 32$, $f(\boldsymbol{x}_{t-1}) = \boldsymbol{x}_{t-1} + h_{t-1} \cdot \frac{d\boldsymbol{x}_{t-1}}{dt}$, $\boldsymbol{w}_t \in \mathbb{R}^{32}$ represents the process noise and we assume that it follows a n-dimensional normal distribution with zero mean and $\boldsymbol{Q}_t \in \mathbb{R}^{32\times32}$ represents covariance matrix of $\boldsymbol{w}_t$.

In terms of observation, daily incidence rates from 12 April 2022 to 15 February 2023 were used as observation data, and the first 19 days were used as a burn-in period for a more precise and stable estimate of β_{ij}. In our age structured transmission model ωE_i represents the theoretical daily incidence, therefore the observation equation is as follows, given that the errors generated during observation.

$$\boldsymbol{y}_t = g(\boldsymbol{x}_t) + \boldsymbol{v}_t, \quad \boldsymbol{v}_t \sim \mathcal{N}_m(\boldsymbol{0}, \boldsymbol{R}_t), \tag{6}$$

where $m = 4$, $g(\boldsymbol{x}_t) = \omega \odot \boldsymbol{E}$ ($\odot$ is the entry-wise product), $\boldsymbol{v}_t \in \mathbb{R}^4$ represents the measurement noise and we assume that it follows a m-dimensional normal distribution with zero mean and $\boldsymbol{R}_t \in \boldsymbol{R}^{4\times4}$ represents the covariance matrix of $\boldsymbol{v}_t$. Equation 5 and Equation 6 together formed a complete description of the disease transmission system as a nonlinear Gaussian state-space model.

An important form of inference for state-space models is filtering, which attempts to sequentially estimate the posterior distribution of the state at the current time point based on all observations collected so far. The work of Geir Evensen *et al.* demonstrates the efficiency of using iterative ensemble filters to estimate the parameters of a SEIR model during the COVID-19 pandemic [31]. In our study, Ensemble Kalman Filter (EnKF) [32] is used to obtain real-time estimations of the state vector $\boldsymbol{x}$. Ensemble Kalman Filter (EnKF) is a filtering method which approximates the Kalman filter by representing the state distribution with an ensemble of draws from that distribution [32]. The EnKF operates through two iterative phases: (1) a forecast step where each ensemble member propagates forward according to the epidemiological model equations, and (2) an analysis step where the ensembles are adjusted based on the discrepancy between predictions and observations, weighted by their respective uncertainties. This approach provides natural uncertainty quantification through the ensemble spread, avoiding restrictive normality assumptions required by traditional Kalman filters. In our study, we used a stochastic EnKF algorithm described in reference [32] and detailed algorithmic procedure is as follows:

Start with an initial ensemble $\hat{\boldsymbol{x}}_0^{(1)}, ..., \hat{\boldsymbol{x}}_0^{(N)}$. Then, at each time $t = 1, 2, ...$, given an ensemble $\hat{\boldsymbol{x}}_{t-1}^{(1)}, ..., \hat{\boldsymbol{x}}_{t-1}^{(N)}$ of draws from the filtering distribution at time $t-1$, the stochastic EnKF carries out the following two steps for $i = 1, ..., N$:

1. **Forecast Step**: Draw $\boldsymbol{w}_t^{(i)} \sim \mathcal{N}_n(\boldsymbol{0}, \boldsymbol{Q}_t)$ and calculate $\tilde{\boldsymbol{x}}_t^{(i)} = f(\hat{\boldsymbol{x}}_{t-1}^{(i)}) + \boldsymbol{w}_t^{(i)}$.
2. **Update Step**: Draw $\boldsymbol{v}_t^{(i)} \sim \mathcal{N}_m(\boldsymbol{0}, \boldsymbol{R}_t)$ and calculate $\hat{\boldsymbol{x}}_t^{(i)} = \tilde{\boldsymbol{x}}_t^{(i)} + \hat{\boldsymbol{K}}_t(\boldsymbol{y}_t + \boldsymbol{v}_t^{(i)} - g(\tilde{\boldsymbol{x}}_t^{(i)}))$, where $\hat{\boldsymbol{K}}_t$ is given in Equation 7.

where $\boldsymbol{y}_t$ represents the observation data we used. The estimated Kalman gain($\hat{\boldsymbol{K}}_t$) has the following form:

$$\hat{\boldsymbol{K}}_t := \boldsymbol{C}_t \boldsymbol{H}_t^T (\boldsymbol{H}_t \boldsymbol{C}_t \boldsymbol{H}_t^T + \boldsymbol{R}_t)^{-1} \tag{7}$$

where $\boldsymbol{C}_t$ represents an estimate of the state forecast covariance matrix $\tilde{\boldsymbol{\Sigma}}_t$. The simplest example is $\boldsymbol{C}_t = \tilde{\boldsymbol{S}}_t$, where $\tilde{\boldsymbol{S}}_t$ is the sample covariance matrix of $\tilde{\boldsymbol{x}}_t^{(1)}, ..., \tilde{\boldsymbol{x}}_t^{(N)}$. $\boldsymbol{H}_t$ represents Jacobian matrices for multivariate vector-valued functions $g(\tilde{\boldsymbol{x}}_t)$.

In our study, the implementation of EnKF used 1000 ensemble members ($N = 1000$) with inflation factor $\lambda = 1.2$ to maintain diversity. Given that observation data are reported once daily, the assimilation frequency is set to one cycle per day. The covariance of measurement noise was calibrated using pre-epidemic baseline data. Due to the stochasticity and uncertainty inherent in both the data and algorithm, We conducted 100 independent runs of the EnKF, generating a distribution of estimates for β_{ij} at each discrete time interval. Figure S4 demonstrates the degree of agreement between the estimates of daily incidence obtained by the EnKF algorithm and the actual reported daily incidence, which indicates that the performance of EnKF is relatively well. Figure S5 presents the distributions of transmission rate coefficients (β_{ij}) across all discrete time intervals estimated by Ensemble Kalman Filter.

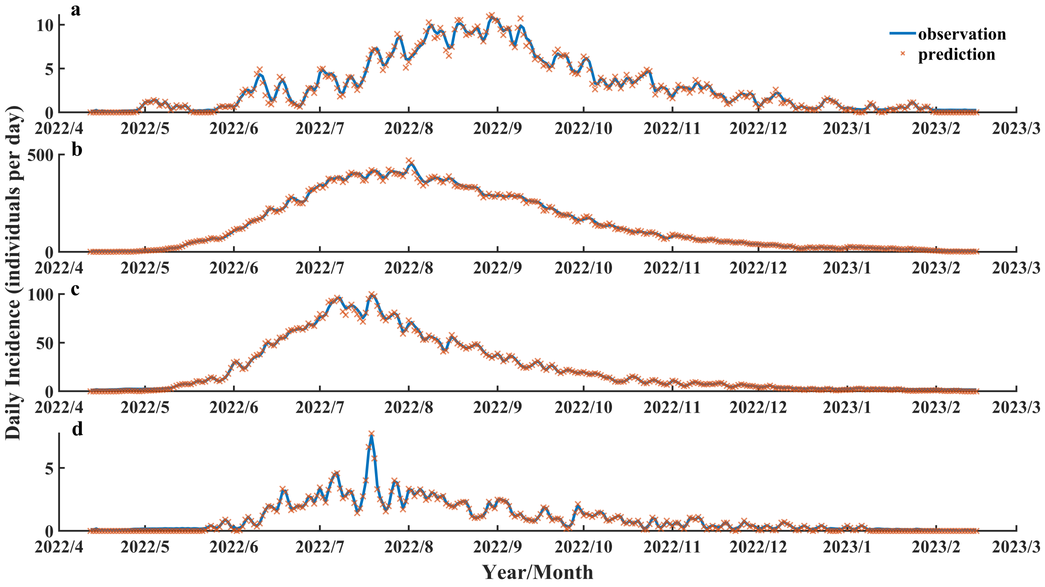

Figure S4: The observation and model prediction. The horizontal axis indicates the date and the vertical axis indicates the number of new cases per day. Subfigures a, b, c, and d represent the four age groups: 0–17, 18–44, 45–64 and 65+ years old respectively. Light red X indicate reported daily incidence and solid blue lines indicate model-predicted daily incidence.

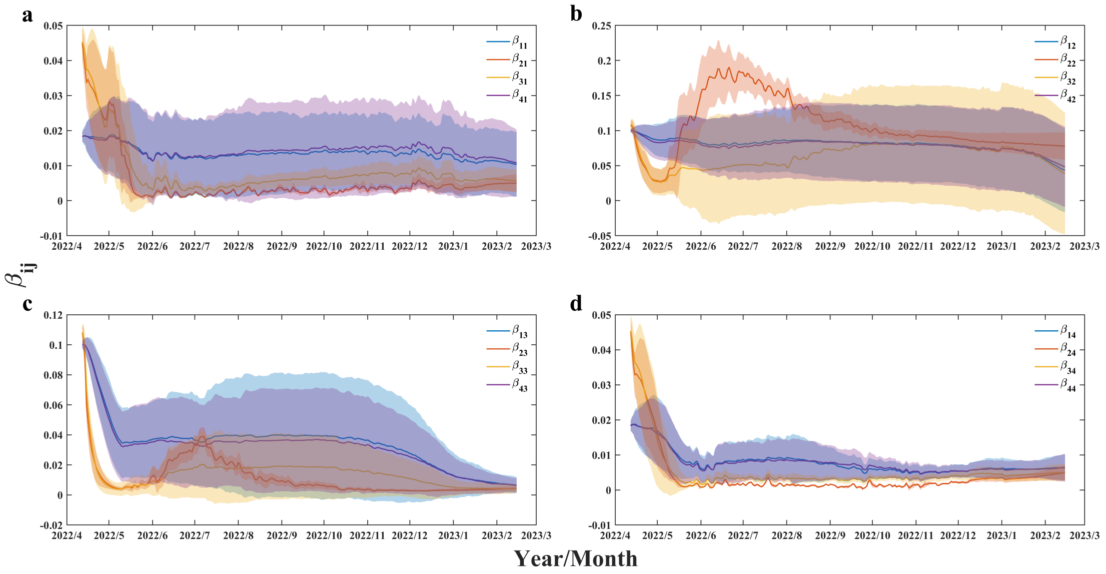

Figure S5: Distributions of transmission rate coefficients (β_{ij}) estimated by EnKF. The horizontal axis indicates the date and the vertical axis indicates the transmission rate coefficient. Subfigures a presents the trends of β_{11}, β_{21}, β_{31} and β_{41} over time. Subfigures b presents the trends of β_{12}, β_{22}, β_{32} and β_{42} over time. Subfigures c presents the trends of β_{13}, β_{23}, β_{33} and β_{43} over time. Subfigures d presents the trends of β_{14}, β_{24}, β_{34} and β_{44} over time. The solid line represents the mean, and the light shaded region represents the 95% credible interval.

4 Effective reproduction number for assessing transmissibility

In general, we use regeneration number to quantify the ability of a disease to spread. The basic reproduction number represents the number of secondary infections produced by an infected person in a fully susceptible population during its infectious stage, while the effective regeneration number represents the number of secondary infections produced by an infected person in a population that is not fully susceptible (e.g., where an intervention has been implemented). Due to emergency response measures implemented by countries worldwide during the 2022 mpox outbreak and the cross-immunization of smallpox vaccine against mpox (with a protection rate of about 85% [33]), total population is not completely susceptible to mpox. As a result, we chose the effective reproduction number to quantitatively assess age-specific transmissibility of the monkeypox virus. The interactive R_{ij} between age groups in our report is defined as the expectation of secondary infections that an infected individual in the age group i will produce in the susceptible population of the age group j during the infectious period. We use R_{eff} to denote the expectation of secondary infections that an infected individual in arbitrary age group will produce in the entire population during its infectious period. We applied the Definition-Based Method (DBM) [34] to compute R_{eff} and the calculation formulas are given by:

$$R_{ij} = \frac{\beta_{ij}}{\gamma}, \tag{8}$$

$$R_{eff} = \sum_{i=1}^{n} \left(\frac{N_i}{N} \sum_{j=1}^{n} R_{ij} \right), \tag{9}$$

where $n = 4$ represents the number of age groups, N_i represents the number of individuals in age group i and N represents the number of individuals in all age groups. Detailed derivation steps and explanation are as follows.

For any infected individual X_i, there are two possible states: E_i, I_i. We consider the initial state of the individual as E_i, and then compute the probability that it develops into I_i:

$$P(X_i \in I_i) = P(X_i \in I_i \mid X_i \in E_i) P(X_i \in E_i) = \frac{\omega}{\omega} = 1 \tag{10}$$

Where $P(X_i \in E_i)$ denotes the probability that individual X_i will develop into the E_i, and $P(X_i \in I_i \mid X_i \in E_i)$ denotes the conditional probability of X_i will develop into I_i with $X_i \in E_i$ known.

The number of secondary cases in age group j produced by X_i per unit time is given by:

$$Q(X_i) = \frac{\beta_{ij} S_j}{N_j} \quad (X_i \in I_i) \tag{11}$$

Generally, if not considering case fatality, the average infectious period of one infected individual in group i is given by:

$$T(X_i) = \frac{1}{\gamma} \quad (X_i \in I_i) \tag{12}$$

It is reasonable to assume that $Q(X_i)$ is constant during the time interval $T(X_i)$. By taking expectations, the expectation of secondary cases in age group j that one

infected individual in age group i will produce during its lifespan as infectious is given by:

$$R_{ij} = P(X_i \in I_i) \times Q(X_i) \times T(X_i) = \frac{\beta_{ij} S_j}{N_j \gamma} \tag{13}$$

Assuming age group j is entirely susceptible, so $S_j = N_j$:

$$R_{ij} = \frac{\beta_{ij}}{\gamma} \tag{14}$$

Let $R(X_i, j)$ denote the random variable describing the secondary cases that individual X_i will produce in age group j during its lifespan as infectious. We define group 0 as the entire population, so $R(X_0, 0)$ denotes the number of secondary infections that one arbitrary infected individual X_0 will produce in the entire population during its lifespan as infectious. We compute the expectation of $R(X_0, 0)$, *i.e.*, R_{eff}:

$$\begin{aligned}
R_{eff} &= \mathbb{E}\left\{R\left(X_0, 0\right)\right\} \\
&= \mathbb{E}\left\{\sum_{j=1}^{n} R\left(X_0, j\right)\right\} \\
&= \sum_{j=1}^{n} \mathbb{E}\left\{R\left(X_0, j\right)\right\} \\
&= \sum_{j=1}^{n} \mathbb{E}_{R(X_i j), i=1,2,\cdots,n}\left\{\mathbb{E}\left\{R\left(X_0, j\right) \mid R\left(X_i, j\right), i = 1, 2, \cdots, n\right\}\right\} \\
&= \sum_{j=1}^{n} \mathbb{E}_{R(X_i, j), i=1,2,\cdots,n}\left\{\sum_{i=1}^{n} \mathbb{P}\left(X_0 \in N_i\right) R\left(X_i, j\right)\right\} \\
&= \sum_{j=1}^{n} \sum_{i=1}^{n} \mathbb{P}\left(X_0 \in N_i\right) \mathbb{E}_{R(X_i, j)}\left\{R\left(X_i, j\right)\right\} \\
&= \sum_{j=1}^{n} \sum_{i=1}^{n} \mathbb{P}\left(X_0 \in N_i\right) R_{ij} \\
&= \sum_{i=1}^{n} \left[\mathbb{P}\left(X_0 \in N_i\right) \sum_{j=1}^{n} R_{ij}\right] \\
&= \sum_{i=1}^{n} \left(\frac{N_i}{N} \sum_{j=1}^{n} R_{ij}\right)
\end{aligned} \tag{15}$$

Here $\mathbb{P}\left(X_0 \in N_i\right)$ denotes the probability of an arbitrary individual X_0 belonging to age group j and it is estimated by $\frac{N_i}{N}$, the proportion of size of age group i. If $R_{eff} < 1$, each infected individual will produce less than one secondary case during the infectious period, and therefore the outbreaks will gradually disappear. If $R_{eff} > 1$, diseases can potentially lead to widespread transmission.

5 Sensitivity analysis

To assess the stability and reliability of the transmission rate coefficients and effective reproduction numbers, we performed a sensitivity analysis for the parameters (incubation and infectious period, initial conditions) in the model. In section 3.1, we have obtained the empirical probability distributions of incubation and infectious period, and we assume that all initial conditions follow uniform distributions within specified ranges. We treat these parameters as distinct dimensions and perform Latin Hypercube Sampling (LHS) according to their respective probability distributions, generating 100 samples (i.e., 100 different combinations of parameters). For each of the 100 parameter combinations, we independently estimated the transmission rate coefficients by EnKF and calculated the corresponding effective reproduction numbers.

The results of the sensitivity analysis are illustrated in Figure S6 and Figure S7. It can be observed that β_{22}, β_{32}, β_{33}, β_{43} and β_{14} exhibit sensitivity to parameter variations, with β_{32} being particularly affected. Furthermore, by holding all other parameters constant while varying a single parameter (or conversely, fixing one parameter while varying the others), we demonstrate that the transmission rate coefficient remains insensitive to changes in both the incubation period and initial conditions, but exhibits marked sensitivity to variations in the infectious period. The effective reproduction number follows a similar pattern: while R_{12}, R_{32}, R_{42}, R_{13}, R_{43} and R_{eff} demonstrate sensitivity to parameter variations, they exhibit relatively lower responsiveness to changes in the infectious period compared to transmission rate coefficients.

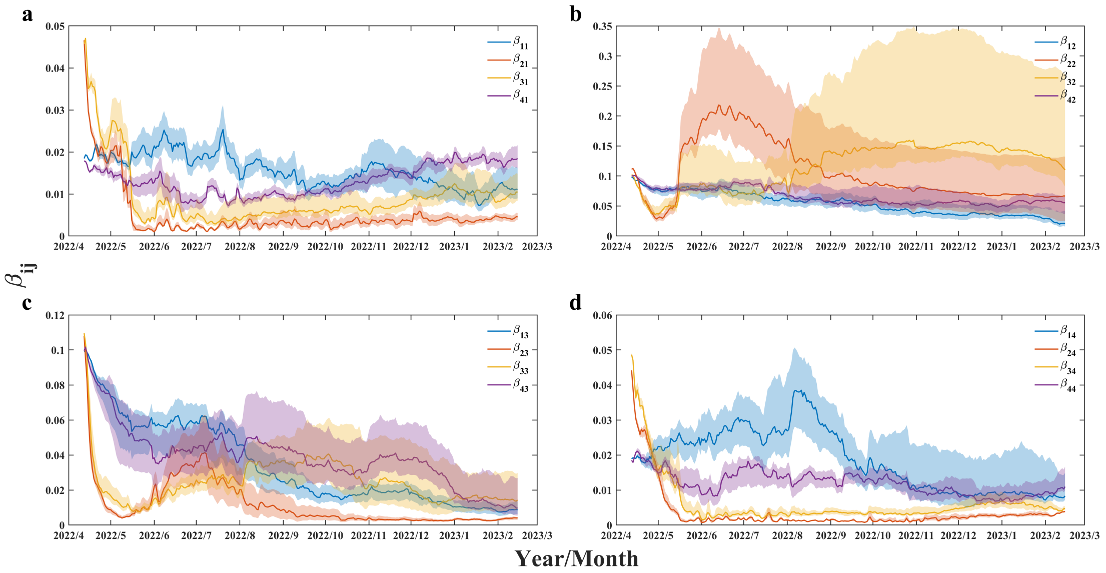

Figure S6: Estimated transmission rate coefficients for different combinations of parameters. Subfigure a illustrates the estimates of β_{11}, β_{21}, β_{31} and β_{41} across all discrete time intervals. Subfigure b illustrates the estimates of β_{12}, β_{22}, β_{32} and β_{42} across all discrete time intervals. Subfigure c illustrates the estimates of β_{13}, β_{23}, β_{33} and β_{43} across all discrete time intervals. Subfigure d illustrates the estimates of β_{14}, β_{24}, β_{34} and β_{44} across all discrete time intervals. The solid line represents the median, and the light shaded region represents the interquartile range.

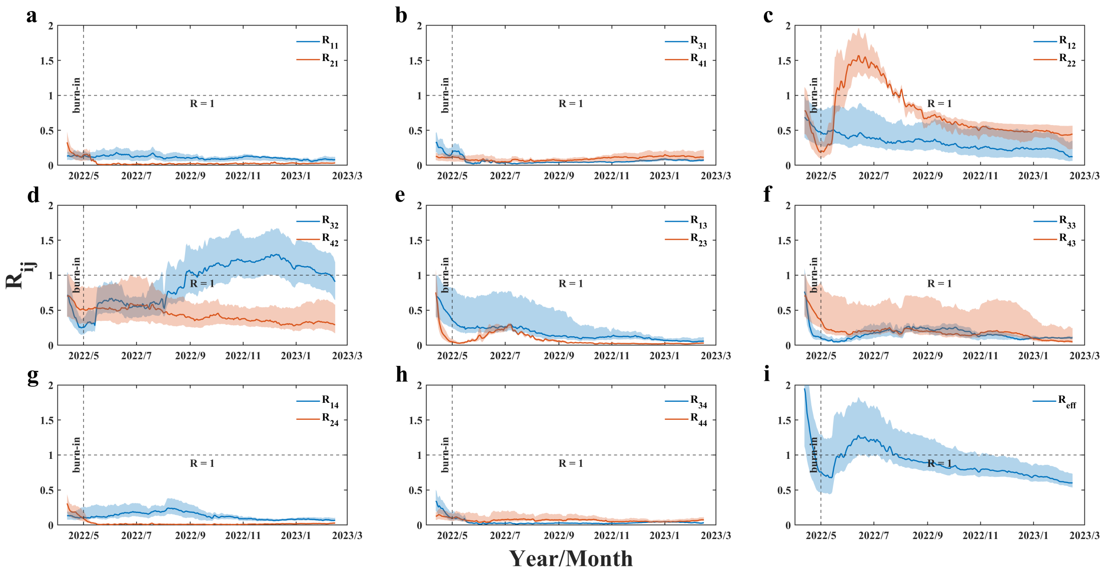

Figure S7: Effective reproduction numbers with or between age groups calculated for different combinations of parameters. Subfigures a to h illustrate the interactive R_{ij} between age groups (the expectation of secondary infections generated by an infected individual from the age group i in the susceptible population of the age group j throughout the infectious period) across all discrete time intervals. Subfigure i illustrates R_{eff} (the expectation of secondary infections that one infected individual will produce in the entire population throughout its infectious period) across all discrete time intervals. The solid line represents the median, and the light shaded region represents the interquartile range. The segment preceding the vertical dashed line indicates the burn-in period, while the horizontal dashed line marks the threshold where the effective reproduction number is equal to 1.

References

[1] *2022-23 mpox outbreak: Global trends*, Web Page, World Health Organization, Geneva, 2023. [Online]. Available: https://worldhealthorg.shinyapps.io/mpx_global/.

[2] *Natural history of disease.* Web Page, Open Access Pub, New York, 2023. [Online]. Available: https://openaccesspub.org/public-health-international/natural-history-of-disease.

[3] A. Khalil, A. Samara, P. O'Brien, *et al.*, "Monkeypox in pregnancy: Update on current outbreak," *Lancet Infect Dis*, vol. 22, no. 11, pp. 1534–1535, 2022.

[4] V. M. Ferré, A. Bachelard, M. Zaidi, *et al.*, "Detection of monkeypox virus in anorectal swabs from asymptomatic men who have sex with men in a sexually transmitted infection screening program in paris, france," *Ann Intern Med*, vol. 175, no. 10, pp. 1491–1492, 2022.

[5] I. De Baetselier, C. Van Dijck, C. Kenyon, *et al.*, "Retrospective detection of asymptomatic monkeypox virus infections among male sexual health clinic attendees in belgium," *Nat Med*, vol. 28, no. 11, pp. 2288–2292, 2022.

[6] D. L. Heymann, M. Szczeniowski, and K. Esteves, "Re-emergence of monkeypox in africa: A review of the past six years," *British Medical Bulletin*, vol. 54, no. 3, pp. 693–702, 1998.

[7] O. Mitja, D. Ogoina, B. K. Titanji, *et al.*, "Monkeypox," *Lancet*, vol. 401, no. 10370, pp. 60–74, 2023.

[8] M. J. Miller, S. Cash-Goldwasser, G. E. Marx, *et al.*, "Severe monkeypox in hospitalized patients - united states, august 10-october 10, 2022," *MMWR Morb Mortal Wkly Rep*, vol. 71, no. 44, pp. 1412–1417, 2022.

[9] F. Miura, C. E. van Ewijk, J. A. Backer, *et al.*, "Estimated incubation period for monkeypox cases confirmed in the netherlands, may 2022," *Euro surveillance*, vol. 27, no. 24, p. 2 200 448, Jun. 2022.

[10] K. Charniga, N. B. Masters, R. B. Slayton, *et al.*, "Estimating the incubation period of monkeypox virus during the 2022 multi-national outbreak," *medRxiv*, Jun. 23, 2022.

[11] J. P. Thornhill, S. Barkati, S. Walmsley, *et al.*, "Monkeypox virus infection in humans across 16 countries - april-june 2022," *N Engl J Med*, vol. 387, no. 8, pp. 679–691, 2022.

[12] A. Català, P. Clavo-Escribano, J. Riera-Monroig, *et al.*, "Monkeypox outbreak in spain: Clinical and epidemiological findings in a prospective cross-sectional study of 185 cases," *The British journal of dermatology*, vol. 187, no. 5, pp. 765–772, Nov. 2022.

[13] E. J. Tarín-Vicente, A. Alemany, M. Agud-Dios, *et al.*, "Clinical presentation and virological assessment of confirmed human monkeypox virus cases in spain: A prospective observational cohort study," *Lancet*, vol. 400, no. 10353, pp. 661–669, 2022.

[14] G. Guzzetta, A. Mammone, F. Ferraro, *et al.*, "Early estimates of monkeypox incubation period, generation time, and reproduction number, italy, may-june 2022," *Emerging infectious diseases*, vol. 28, no. 10, pp. 2078–2081, Oct. 2022.

[15] M. Mailhe, A.-L. Beaumont, M. Thy, *et al.*, "Clinical characteristics of ambulatory and hospitalized patients with monkeypox virus infection: An observational cohort study," *Clinical microbiology and infection : the official publication of the European Society of Clinical Microbiology and Infectious Diseases*, vol. 29, no. 2, pp. 233–239, Feb. 2023.

[16] D. Moschese, G. Pozza, A. Giacomelli, *et al.*, "Natural history of human monkeypox in individuals attending a sexual health clinic in milan, italy," *The Journal of infection*, vol. 86, no. 1, e18–e20, Jan. 2023.

[17] M. Gomez-Garberi, P. Sarrio-Sanz, L. Martinez-Cayuelas, *et al.*, "Genitourinary lesions due to monkeypox," *European urology*, vol. 82, no. 6, pp. 625–630, Dec. 2022.

[18] K. O'Laughlin, F. A. Tobolowsky, R. Elmor, *et al.*, "Clinical use of tecovirimat (tpoxx) for treatment of monkeypox under an investigational new drug protocol - united states, may-august 2022," *MMWR. Morbidity and mortality weekly report*, vol. 71, no. 37, pp. 1190–1195, Sep. 16, 2022.

[19] K. M. Angelo, T. Smith, D. Camprubí-Ferrer, *et al.*, "Epidemiological and clinical characteristics of patients with monkeypox in the geosentinel network: A cross-sectional study," *The Lancet. Infectious diseases*, vol. 23, no. 2, pp. 196–206, Feb. 2023.

[20] Z. J. Madewell, K. Charniga, N. B. Masters, *et al.*, "Serial interval and incubation period estimates of monkeypox virus infection in 12 jurisdictions, united states, may-august 2022," *Emerging infectious diseases*, vol. 29, no. 4, pp. 818–821, Apr. 2023.

[21] T. Ward, R. Christie, R. S. Paton, F. Cumming, and C. E. Overton, "Transmission dynamics of monkeypox in the united kingdom: Contact tracing study," *BMJ (Clinical research ed.)*, vol. 379, e073153, Nov. 2, 2022.

[22] C. Besombes, F. Mbrenga, L. Schaeffer, *et al.*, "National monkeypox surveillance, central african republic, 2001-2021," *Emerging infectious diseases*, vol. 28, no. 12, pp. 2435–2445, Dec. 2022.

[23] S. T. Kröger, M. C. Lehmann, M. Treutlein, *et al.*, "Mpox outbreak 2022: An overview of all cases reported to the cologne health department," *Infection*, 10.1007/s15010–023-01997-x, Feb. 14, 2023.

[24] *Mpox(monkeypox)*, Web Page, World Health Organization, Geneva, 2023. [Online]. Available: https://www.who.int/news-room/fact-sheets/detail/monkeypox.

[25] A. B. Al-Tammemi, R. Albakri, and S. Alabsi, "The outbreak of human monkeypox in 2022: A changing epidemiology or an impending aftereffect of smallpox eradication?" *Frontiers in Tropical Diseases*, vol. 3, 2022.

[26] D. Kmiec and F. Kirchhoff, "Monkeypox: A new threat?" *International journal of molecular sciences*, vol. 23, no. 14, p. 7866, Jul. 17, 2022.

[27] A. Apte, "An introduction to data assimilation," in *Applied Mathematics*, S. Sarkar, U. Basu, and S. De, Eds., New Delhi: Springer India, 2015, pp. 31–42.

[28] N. Chopin and O. Papaspiliopoulos, "Introduction to state-space models," in *An Introduction to Sequential Monte Carlo*. Cham: Springer International Publishing, 2020, pp. 11–25.

[29] D. Ogaz, Q. Enayat, J. R. G. Brown, *et al.*, "Mpox diagnosis, behavioral risk modification, and vaccination uptake among gay, bisexual, and other men who have sex with men, united kingdom, 2022," *Emerging infectious diseases*, vol. 30, no. 5, pp. 916–925, May 2024.

[30] X.-S. Zhang, S. Mandal, H. Mohammed, *et al.*, "Transmission dynamics and effect of control measures on the 2022 outbreak of mpox among gay, bisexual, and other men who have sex with men in england: A mathematical modelling study," *The Lancet Infectious Diseases*, vol. 24, no. 1, pp. 65–74, 2024.

[31] G. Evensen, J. Amezcua, M. Bocquet, *et al.*, "An international initiative of predicting the sars-cov-2 pandemic using ensemble data assimilation," *Foundations of Data Science*, vol. 3, no. 3, pp. 413–477, 2021.

[32] M. Katzfuss, J. R. Stroud, and C. K. Wikle, "Understanding the ensemble kalman filter," *The American Statistician*, vol. 70, no. 4, pp. 350–357, 2016.

[33] G. A. Poland, R. B. Kennedy, and P. K. Tosh, "Prevention of monkeypox with vaccines: A rapid review," *The Lancet Infectious Diseases*, vol. 22, no. 12, e349–e358, 2022.

[34] X. Guo, Y. Guo, Z. Zhao, *et al.*, "Computing r (0) of dynamic models by a definition-based method," *Infect Dis Model*, vol. 7, no. 2, pp. 196–210, 2022.
